# Does the Type of Online Group-based Exercise Influence the Feasibility of Telerehabilitation in People with Parkinson’s Disease? A Randomized Controlled Trial

**DOI:** 10.63144/ijt.2026.6748

**Published:** 2026-06-01

**Authors:** Francisca Pereira, Camila Pinto, Rafaela Simon Myra, Thainara Cruz da Rosa, Ana Paula da Silva, Bruna Medeiros Calegare, Bruno Rodrigues da Silva, Lívia Estivalet Vieira, Aline Souza Pagnussat

**Affiliations:** 1 Graduate Program in Health Sciences, Universidade Federal de Ciências da Saúde de Porto Alegre (UFCSPA), Porto Alegre, Rio Grande do Sul, Brazil; 2 Movement Analysis and Rehabilitation Laboratory, Universidade Federal de Ciências da Saúde de Porto Alegre, Porto Alegre, Rio Grande do Sul, Brazil; 3 Department of Physical Therapy, Universidade Federal do Rio Grande do Sul (UFRGS), Porto Alegre, Rio Grande do Sul, Brazil; 4 Graduate Program in Rehabilitation Sciences, Universidade Federal de Ciências da Saúde de Porto Alegre (UFCSPA), Porto Alegre, Rio Grande do Sul, Brazil

**Keywords:** Dance therapy, Exercise therapy, Feasibility studies, Parkinson’s disease, Telerehabilitation

## Abstract

**Background:**

Ensuring equitable access to effective interventions for people with Parkinson’s disease (PD) remains a global challenge. Although dance and multimodal exercise improve motor and non-motor symptoms, no randomized controlled trial has compared their feasibility, safety, and participants’ perceptions when delivered online in a group-based format.

**Methods:**

This randomized controlled feasibility trial allocated participants to a dance or multimodal exercise group incorporating music. Interventions were delivered via videoconference twice weekly for 12 weeks. Feasibility and safety were assessed through adherence, attendance, technological barriers, and adverse events. Participant perceptions were evaluated post-intervention using study-specific structured interviews.

**Results:**

Seventy-five participants were enrolled (dance: n = 40; multimodal exercise: n = 35), predominantly women, with mild-to-moderate PD (Hoehn & Yahr Scale 1.0–3.0). Both interventions were feasible and safe, with high adherence (82.5–85.7%), moderate attendance, and no adverse events with similar participants’ perceptions across the groups.

**Conclusions:**

Group-based online dance and multimodal exercise are feasible, safe, and well accepted for people with mild-to-moderate PD, supporting their use as telerehabilitation options in clinical practice.

The heterogeneity of symptoms in people with Parkinson’s disease (PD) has led to an increased need for diverse individualized treatment approaches (Leite Silva et al., 2023; Nilsson et al., 2015). However, common barriers include transportation difficulties due to mobility limitations, high healthcare costs, low engagement, and limited access to specialized services (Akgün et al., 2025; Shahriar Zaman et al., 2021). In response to the COVID-19 pandemic, online exercise programs increased physical activity levels and promoted equitable treatment access (Colón-Semenza et al., 2024; Keteyian et al., 2022; Morris et al., 2021; Pinto et al., 2023; Tardelli et al., 2023). Telerehabilitation and group-based programs may represent potential strategies to overcome barriers. Telerehabilitation for people with PD requires further investigation with larger samples and rigorous randomized controlled designs to support its implementation in clinical practice.

Dance and multimodal exercise represent distinct evidence-based interventions commonly used in clinical and community settings to manage PD symptoms (Osborne et al., 2022). Dance has been shown to reduce motor symptoms, improve balance and functional mobility, and reduce depressive symptoms, whereas multimodal exercise may reduce motor disease severity and improve quality of life (Carpellate et al., 2020; Ismail et al., 2021). Multimodal exercise is structured around sets and repetitions and involves aerobic exercise, strength, balance, and functional tasks (Ernst et al., 2023; Padilha et al., 2023). Dance uses choreographed or improvised movements with music and integrates rhythm, coordination, sequence memory, and emotional expression (dos Santos Duarte et al., 2023; Pinto et al., 2023). Although both interventions can be delivered via telerehabilitation, differences in structure, motor demands, and supervision may influence feasibility, safety, and participant experience.

In the context of telerehabilitation, little is known about how these interventions perform, particularly in contexts where physical risks may be increased, and direct physical assistance is limited. Therefore, evaluating participants’ perceptions and potential barriers is essential to inform the implementation of safe and acceptable intervention modalities for telerehabilitation in clinical practice (Cools et al., 2025). This study aimed to evaluate and compare the feasibility, safety, and participant perceptions of an online dance program and an online multimodal exercise program for people with PD.

## Methods

### Ethics Approval and Registration

This study was registered at ClinicalTrials.gov (Protocol ID: NCT05910216). The study followed the CONSORT (Consolidated Standards of Reporting Trials) extension for feasibility trials statement for reporting results (Eldridge et al., 2016). The study protocol was approved by the Research Ethics Committee of the Universidade Federal de Ciências da Saúde de Porto Alegre (No. 6.301.330) on September 14, 2023. This study reports a pre-specified feasibility analysis from a randomized controlled trial described in a previously published protocol (Pinto et al., 2024). All participants provided written informed consent.

### Participants and Eligibility Criteria

Participants aged between 40–90 years were eligible if they had a diagnosis of PD confirmed by a qualified professional, scored at least 17 points on the Telephone Montreal Cognitive Assessment (T-MoCA) (Katz et al., 2021), and had stable medication and exercise routines for at least four weeks prior to study onset. All participants were required to provide medical documentation confirming their diagnosis. Participants were excluded if they had conditions that could interfere with participation, such as significant auditory and/or visual impairments, other neurological diseases, and/or severe neuromuscular and/or cardiopulmonary diseases, which could hinder participation and understanding of the activities. Participants were required to have access to an internet-connected device and be able to participate in online sessions. For participants who had more severe PD impairment (Hoehn and Yahr scale > 2.5), a caregiver or family member was required to be present during both assessments and intervention sessions (Goetz et al., 2004).

**Figure 1:**
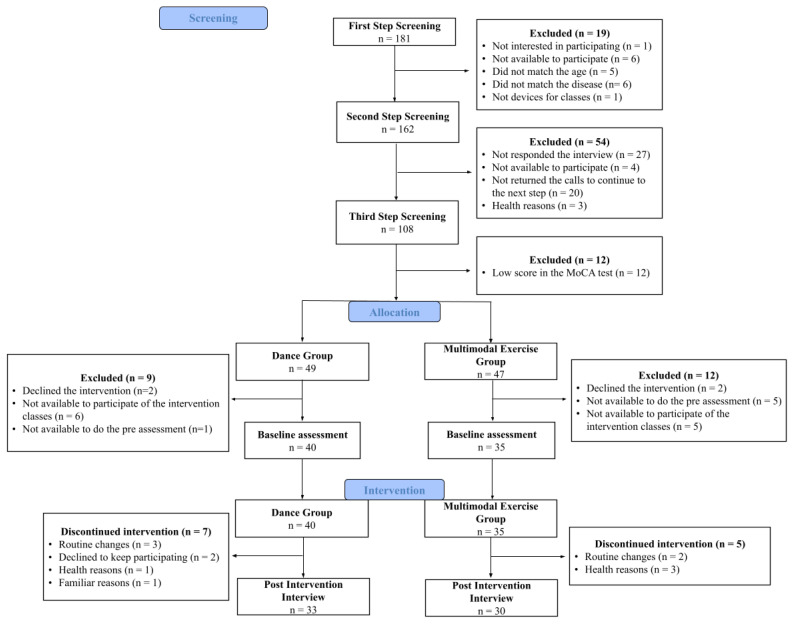
Flow Diagram of the Study

Recruitment was conducted remotely using digital posters. As a first step screening, people who expressed interest were directed to an initial online survey to provide demographic information. For the second step screening, another survey was sent to determine the presence of comorbidities, health conditions, diagnosis, functional difficulties, presence of deep brain stimulation (DBS), Lawton and Brody scale scores (Bouça-Machado et al., 2022), and the availability of devices for participation in the intervention. Subsequently, potential participants were invited to respond to the T-MoCA to verify eligibility (Katz et al., 2021). Those who scored above 17 points were invited to participate in an online assessment via Zoom conducted by a blinded assessor. During this assessment physical activity level, disease stage, freezing of gait, medication use and schedule, and previous experience and perceptions of dance were evaluated using the short version of International Physical Activity Questionnaire (Short-IPAQ) (Lee et al., 2011), the Hoehn & Yahr (H&Y) scale based on a prior in-person evaluation (Goetz et al., 2004), the third item of the New Freezing of Gait Questionnaire (N-FOG) (Nieuw Boer et al., 2009), and the Goldsmiths Dance Sophistication Index (Gold-DSI) (Pinto et al., 2025) respectively. As a third step screening, during the same session, people who met the eligibility criteria received the Informed Consent Form and Image Authorization Form to read and sign. Participants were instructed to maintain their dopaminergic medication and physical activity levels without modifications throughout the study. All assessments were conducted during the ON medication state. No participants received financial compensation. A flowchart of participant inclusion is presented in Figure 1.

All participants received tutorials on how to use the platform and organize their space. They also had direct contact with the research team to request assistance during the interventions and, if necessary, during the assessments.

### Randomization

Participants were randomly allocated in a 1:1 ratio to either a dance with music group or a multimodal exercise with music group. Participants were stratified by H&Y scores. Block randomization was performed by a blinded researcher using Microsoft Excel to ensure balanced group sizes.

Participants were instructed not to reveal their group allocation to the blinded assessor involved in the study. The assessor remained blinded to group allocation and was not involved in any study procedures other than outcome assessments. Blinding of therapists and participants was not feasible due to the nature of the interventions.

### Outcomes Assessments

The domains of feasibility, safety, and participants’ perceptions were evaluated using two approaches:

- Primary outcomes: Researchers’ observations during live sessions, including adherence, attendance, technological barriers, and safety.
- Secondary outcomes: Participants’ perceptions following intervention, including perceived feasibility, enjoyment, engagement, and socialization, were assessed through a study-specific structured interview as described in our published protocol (Pinto et al., 2024). The interview was developed by the research team for the purposes of this study and did not undergo formal validation.

The adherence, attendance, technological barriers, and safety were analyzed based on a previous feasibility study that included recorded classes (Pinto et al., 2023). These outcomes were assessed through the researchers’ observations during the intervention sessions. The research team consisted of four assistants and one instructor per intervention. All data were recorded in a spreadsheet for analysis.

Participants’ perceptions were assessed using a structured interview that included questions on perceived feasibility, enjoyment, engagement, and socialization. The interview was conducted post-intervention by a blinded researcher who had no contact with participants during the intervention sessions.

### Outcomes

#### Feasibility

**Figure 2:**
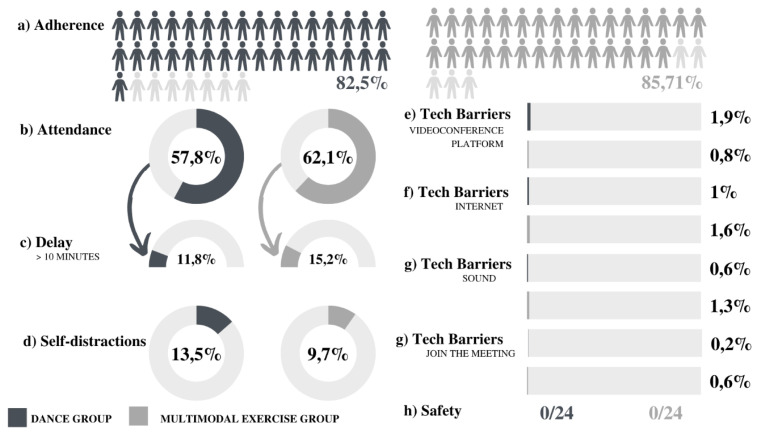
Feasibility Outcomes of the Interventions

The outcomes related to feasibility were calculated as rates, as presented in Figure 2, based on a previous study (Pinto et al., 2023). Adherence was defined as the percentage of participants who completed the intervention. Attendance was calculated as the percentage of sessions attended. Technological barriers were identified based on difficulties reported by participants related to the Zoom platform, including issues with video connection, audio, and internet stability.

#### Safety

Safety was assessed based on the occurrence of adverse events during the intervention sessions and expressed as absolute counts. A total of 24 sessions per intervention was considered.

#### Participants’ Perceptions

Participants’ perceptions were assessed using a structured interview. The interview examined three domains: (1) Feasibility (questions 1–14); (2) Enjoyment and Engagement (questions 15–33); and (3) Socialization (questions 34–35). Responses were scored on a 0–5 Likert scale ranging from strongly agree to strongly disagree, with higher scores indicating worse perceptions. The total score ranged from 0 to 120.

### Intervention Groups

Interventions were conducted twice weekly for 12 weeks, with each session lasting one hour. Full details are available in the published protocol following the Template for Intervention Description and Replication (Tidier) (Hoffmann et al., 2014; Pinto et al., 2024).

***Group 1*** participated in an online, group-based telerehabilitation program incorporating dance and music, inspired by the *Dance for Parkinson’s* (USA)® approach. The intervention emphasized artistic and embodied motor learning strategies, including imagery, environmental cues, and analogy-based learning to support movement execution. Sessions involved the collaborative creation of movement sequences and choreographic structures, integrating improvisation, dance-based movements, rhythmic patterns, and music-related motor responses.

***Group 2*** participated in an online, group-based telerehabilitation program centered on multimodal therapeutic exercise combined with music, designed in accordance with current scientific evidence and the Physical Therapy Guidelines for People with Parkinson’s Disease (Osborne et al., 2022). The program consisted of predefined exercise routines performed in standardized sets and repetitions. In this condition, music was used solely as background accompaniment and not as a tool for motor cueing or facilitation.

Both interventions were delivered by physical therapists with expertise in Parkinson’s disease who were independent of the research team. Sessions included verbal instructions and visual demonstrations to support participant autonomy, including choice, independent decision-making, and self-directed learning. Therapists repeatedly modeled the movements throughout each session to facilitate observation and motor replication. Musical selections were tailored to participants’ preferences to enhance engagement and motivation.

To support feasibility and adherence, participants received structured reminders throughout the intervention period. A group message was sent one day before each session, followed by a session-specific reminder containing the online access link on the day of the intervention, delivered via messaging applications and email. Research assistants attended all sessions to monitor attendance, manage technical aspects, and provide real-time support. Participants without prior experience using Zoom received individualized training before the intervention.

### Sample Size

A minimum of 62 individuals (31 per group) was required, considering an alpha level of 0.05, a power of 80%, and an anticipated dropout rate of up to 20%, to detect differences between the groups in the total structured interview score for perceived feasibility, based on preliminary data from our study.

### Statistical Analysis

The Shapiro-Wilk Test was used to verify data normality, and Levene’s Test was used to verify the homogeneity of variances. Categorical variables were presented as frequencies and percentages, while continuous variables were expressed as mean and standard deviation or median and interquartile range, as appropriate. Group comparisons were performed using the Chi-square test, Fisher’s Exact test, and generalized linear models according to data distribution and sample size. Analyses were performed using SPSS statistical software (IBM SPSS Statistics for Windows, Version 25.0. Armonk, NY: IBM Corp.). The significance level was set at 0.05.

## Results

Seventy-five participants began the intervention (dance: n = 40; multimodal exercise: n = 35), as shown in Figure 1. Randomization was performed prior to baseline assessment due to the group-based nature of the interventions, which required predefined group allocation for scheduling and simultaneous session initiation. Consequently, some randomized participants did not complete the baseline assessment, primarily due to withdrawal or scheduling constraints, resulting in unequal final group sizes despite the initial 1:1 allocation. Despite this approach, groups remained comparable at baseline across key variables.

Feasibility (i.e., adherence, attendance, and technological barriers) and safety were monitored across 24 sessions in both groups. All participants who initiated the intervention were included in the feasibility analyses. Participants’ perceptions (i.e., perceived feasibility, enjoyment, engagement, and socialization) were assessed post-intervention using structured interviews. Of the 75 participants, 63 completed the post-intervention assessment (dance: n = 33; multimodal exercise: n = 30). Analyses of participants’ perceptions were conducted using complete-case data. Feasibility outcomes were calculated based on the full sample of participants who initiated the intervention (n = 75). Participants who did not complete the post-intervention assessment were contacted and encouraged to participate. Reported reasons for non-attendance included scheduling constraints, health-related issues, and lack of availability.

Baseline characteristics are presented in Table 1. The groups were similar in terms of age, disease severity, physical activity levels, dance perception and experience, functional profile, and PD-related characteristics. The mean age was 58.7±8.6 years in the dance group and 60.2±8.7 in the multimodal exercise group. Most participants presented a mild-to-moderate PD, with H&Y stages 2.0 (76.6%) and 3.0 (45.7%) predominant. The use of DBS was low in our sample. Participants in both groups were able to walk independently indoors and outdoors, presented FOG, and were predominantly white women from urban areas. The multimodal exercise group showed higher physical activity levels, whereas the dance group presented moderate levels. Dance experience was similar between groups.

### Feasibility

Feasibility outcomes are presented as percentages in Figure 2. The dance group is presented in dark gray, and the multimodal exercise group in light gray. Overall, feasibility indicators included adherence, attendance, session delays, and participant-related distractions. Adherence was 82.5% in the dance group and 85.71% in the multimodal exercise group. Attendance was 57.8% and 62.1%, respectively. Session delays occurred in 11.8% of dance group sessions and 15.2% of multimodal exercise sessions.

Figure 2 represents the feasibility outcomes observed during sessions from the dance group (n= 40) and multimodal exercise group (n= 35) during the whole intervention period (totaling 24 sessions). On the figure, (a) Adherence (%) calculated from the formula [=total number of individuals who completed the full intervention/the total number of individuals included at the beginning]*100%; (b) attendance (%), calculated as [=number of sessions attended/24]*100, expressed as an average (%); (c) delay of more than 10 minutes in joining the session (%), calculated as [=number of times delayed/number of sessions attended]*100, expressed as an average; (d) self-distractions (%), calculated from the formula [=distractions/number of sessions attended]*100; expressed as an average;(e) tech barriers related to the videoconference platform (%) calculated as [=number of support needed by the researcher’s team/24/total number of participants]*100; (f) tech barriers related to internet and connection problems (%) calculated as [=number of internet problems/24/total number of participants]*100; (g) tech barriers related to the sound (%) were calculated as [=participant reports/24/total number of participants]*100; and (h) safety was reported as [=number of adverse events during the interventions/total number of sessions (24)].

Participant-related distractions occurred in 13.5% of dance sessions and 9.7% of multimodal exercise sessions, most commonly mobile phone use, conversations, or loss of attention. Requests for technical assistance occurred in 1.9% of dance sessions, mainly related to videoconferencing management. Additional barriers, primarily related to internet connectivity, were reported in 1.0% and 1.6% of sessions, respectively. Difficulties joining sessions occurred in 0.2% of dance sessions and 0.6% of multimodal exercise sessions. In the multimodal exercise group, four participants remained seated throughout at least one session; two did so once, one in four sessions, and one in seven sessions. In the dance group, six participants remained seated for at least one session; three did so twice and three once.

### Safety

No adverse events related to the intervention were observed in either group. The interventions were well tolerated. One episode of anxiety was reported by a participant in the dance group during the study period; however, it was not considered related to the intervention, as symptoms were present prior to session participation.

### Participants’ Perceptions

Participants’ perceptions are detailed in Table 2. Results are reported as percentages reflecting the most frequent responses. Most participants accessed the intervention using mobile phones, participated alone, and reported no difficulties related to connectivity, audio, or visual aspects of the sessions. A high perception of safety was reported across both groups. Overall, there was no difference between the groups related to perceived feasibility, enjoyment, engagement, socialization domains, or total final interview score. In question 3.1, related to the difficulties encountered during the interventions, participants reported having no experience with the platform, fear of making mistakes, difficulties managing technology, as well as memory and vision challenges.

## Discussion

This study aimed to evaluate and compare the feasibility, safety, and participants’ perceptions of online dance and online multimodal exercise programs for people with Parkinson’s disease. Our findings indicate that both interventions are feasible and safe for individuals with mild-to-moderate PD, with high adherence, moderate attendance, and similar participants’ perceptions across groups. These results are consistent with previous studies (Jarvis et al., 2022; Lee et al., 2025; Morris et al., 2021; Pinto et al., 2023) and support the use of telerehabilitation as an alternative strategy to promote and maintain physical activity. Both interventions were well accepted, with comparable levels of enjoyment and socialization, suggesting that different exercise modalities can be effectively delivered in a group-based online format.

Online exercise delivery, however, presents specific challenges compared to in-person interventions. These include difficulties in understanding and executing movements effectively, the need to attend to additional factors such as screen-based interaction, device handling, and internet connectivity, as well as potential reduction in in-person social support (Goldman et al., 2023; Keteyian et al., 2022; Peacock et al., 2020). All these factors may have impacted motivation and attendance. Additionally, people with PD present a wide range of motor and non-motor symptoms such as rigidity, tremor, bradykinesia, anxiety, and depression, which may interfere with the use of digital devices, including computers, mobile phones, and tablets (Laar et al., 2023). Other barriers to accessing telerehabilitation include pain, reduced motor skills, low physical fitness, and fatigue (Manago et al., 2021). Consistent with previous studies, some participants in the present study reported difficulties managing digital devices and limited experience with the online platform, despite receiving structured support and instructional tutorials. These findings point to the importance of providing continuous social and technical support, as well as tailoring interventions to participants’ needs and preferences to enhance comfort and enjoyment (Goldman et al., 2023; Laar et al., 2023; Morris et al., 2021).

Although adherence was high, attendance in both interventions was moderate, considering all participants who initiated the intervention. These findings differ from studies conducted during the COVID-19 pandemic, when social restrictions increased participation in online activities (Bek et al., 2021; Colón-Semenza et al., 2024; Lima et al., 2022; Manago et al., 2021; Pinto et al., 2023). In the present study, participants reported difficulties integrating scheduled online sessions into their daily routines, with health-related issues and routine demands being the most common reasons for non-attendance. Additional barriers included challenges with technology use, preference for in-person interaction, and the presence of freezing of gait, which may increase fall risk and negatively affect participation (Manago et al., 2021; Peacock et al., 2020). From a clinical perspective, these findings emphasize the importance of flexible scheduling and consideration of medication cycles, particularly for individuals experiencing off-medication periods. Strategies such as structured team support, group-based supervision, and predefined safety procedures (e.g., caregiver contact information, emergency protocols, and recommendations for supervision during more symptomatic periods) may contribute to increased perceived support and safety, although they may not fully overcome scheduling-related barriers.

Differences in participants’ ability to hear and follow instructor cues between interventions highlight the importance of careful program design in online settings. In the multimodal exercise group, instructions were delivered with background music, which may have interfered with the clarity of verbal cues. In contrast, in the dance group, instructions were typically provided without music, potentially facilitating comprehension. These findings suggest that delivering instructions in silence, followed by the introduction of music after movement explanation, may improve understanding and execution of movements in telerehabilitation contexts. Clear verbal communication, appropriate use of music, and consistent visual demonstration appear to be key elements for optimizing motor learning and engagement in online interventions. Despite their structural differences, both interventions share key components such as motor practice, auditory stimulation, and socialization, which may have contributed to the similar feasibility and participant perceptions observed

Participants in both groups reported perceived improvements in balance, memory, mood, sleep quality, and socialization. Both interventions may provide distinct benefits. Participants in the dance group reported greater perceived improvements in mood, suggesting that dance-based interventions may be particularly suitable for individuals experiencing reduced motivation, depressive symptoms, or social isolation. The integration of music, rhythm, and emotional expression may enhance engagement and enjoyment in this population (Cheng et al., 2024; dos Santos Duarte et al., 2023; Fong Yan et al., 2024; Yang et al., 2022). Dance-based approaches may enhance cognitive and psychosocial processes through choreographic learning (Liu et al., 2021; Yuan et al., 2022), while multimodal exercise may support physical function and functional performance through structured, task-oriented training (Ferrosol-Pastrana et al., 2023; Gamborg et al., 2022). Taken together, these results underscore the importance of considering individual preferences when selecting interventions for people with PD, as alignment with personal goals may improve adherence, enjoyment, and overall satisfaction (Cools et al., 2025; Li et al., 2024).

### Limitations

This study has some limitations. Participants predominantly presented mild-to-moderate PD and moderate-to-high physical activity levels, which may limit the generalizability of the findings to individuals with more advanced disease or lower baseline function. In addition, the sample was predominantly composed of women, although PD is more prevalent in men (Powe et al., 2017). This may reflect higher participation rates among women in healthcare programs, which may explain the predominance of women in our study (Fullard et al., 2018). This aspect limits the generalizability of our findings, supporting the importance of future studies with different sample profiles. Both groups presented similar levels of prior dance experience, which may have influenced participants’ familiarity with movement-based activities. Finally, although the study followed a previously published protocol (Pinto et al., 2024) and included a larger sample than previous studies (Osborne et al., 2022), the feasibility design limits conclusions regarding effectiveness, and the structured interview was not formally validated, which may limit the interpretability of subjective outcomes.

## Conclusions

This feasibility study indicates that online dance and multimodal exercise are feasible, safe, and well accepted by individuals with mild-to-moderate PD. Both interventions demonstrated high adherence, moderate attendance, and similar participants’ perceptions across the groups. Participants reported perceived improvements in aspects such as mood, sleep quality, balance, memory, and socialization; however, these findings reflect subjective perceptions rather than objectively measured outcomes. The sample characteristics, including the predominance of women, access to digital resources, and moderate-to-high physical activity levels, may limit the generalizability of the findings. These results support the use of group-based telerehabilitation as a complementary strategy to promote physical activity and expand access to care for people with PD.

## Figures and Tables

**Table 1:** Descriptive Characteristics at Baseline

	Dance Group (n =40)	Multimodal Exercise Group (n= 35)	*p-valor*
**General Characteristics**			

Age *(years*)	58.7 ± 8.6	60.2 ± 8.7	0.442
Body Mass (*Kg*)	68.2 ± 12.3	68.6 ± 12.1	0.906
BMI *(Kg/m^2^)*	25.9 ± 4.3	26.3 ± 4.4	0.739
Gender *(F/M)*	(34/6)	(30/5)	1.000
Ethnicity (*White/Black/Mixed race/Indigenous)*	(30/4/6/0)	(27/1/6/1)	0.451
Educational Level (*incomplete primary/primary/secondary/tertiary)*	(1/1/13/25)	(0/1/12/22)	0.499
Location of domicile (*urban/rural)*	(39/1)	(33/2)	
T-MoCA *(0–22)*	19.2 ± 1.5	19.5 ± 1.8	0.464

**PD Clinical Characteristics**			

Hoehn & Yahr *(1.0–2.5/3.0/4.0)*	(31/8/1)	(26/9/0)	0.559
DBS *(Y/N)*	(5/35)	(2/33)	0.438
Presence of FOG *(Y/N)*	(25/15)	(20/15)	0.813
PD Diagnosis *(Years)*	8 [4.5 – 11]	6 [4 – 9]	0.203
PD Symptoms *(Years)*	12 [6.5 – 14]	9 [6 – 13]	0.289
LEDD *(mg)*	862.6 ± 514,7	898.7 ± 610	0.782

**Mobility Parameters**			

Lawton scale (*8–24)*	23 [21.5 – 24]	23 [21 – 24]	0.804
Falls - last 12 months *(Y/N)*	(23/17)	(15/20)	
Walk - Inside Home (*device/supervision/independently)*	(6/1/33)	(2/4/29)	0.154
Walk - Outside Home (*device/supervision/independently)*	(4/7/29)	(3/10/22)	0.521
Short IPAQ *(high/moderate/low)*	(17/19/4)	(21/9/5)	0.151

**Dance Experience**			

Gold-DSI *(1–7)*	4.17 ± 0.82	4.13 ± 0.73	0.810

*Note*. Data are presented as the number of participants, mean ± standard deviation or median (interquartile range), depending on data distribution. P-value < 0.05 was considered statistically significant.

*Abbreviations:* BMI, Body Mass Index; T-MoCA, Telephone Montreal cognitive assessment; DBS, Deep Brain Stimulation; FOG, Freezing of Gait; PD, Parkinson’s Disease; LEDD, Levodopa Equivalent Daily Dose; IPAQ, International Physical Activity Questionnaire; Gold-DSI, Goldsmiths Dance Sophistication Index.

**Table 2:** Participant’s Perception - Structured Interview Results (n = 63)

	Dance Group (n= 33)		Multimodal Exercise Group (n= 30)		Group Comparison

Questions	Recurrent answer	Frequency	Recurrent answer	Frequency	*p-value*
**SECTION I - FEASIBILITY**					

1. I needed assistance from someone to access the class link (family/caregiver/friend/research team).	No	72.7%	No	63.3%	0.840
2. ^*^Which device(s) did you use during the classes?	Cell Phone	57.5%	Cell Phone	70.0%	0.447
3. I found managing the devices to participate in the classes difficult.	Strongly Disagree	66.7%	Strongly Disagree	66.6%	
3.1 If your answer is ‘agree,’ please detail the difficulties you encountered:^*^	Difficulty related to technology management	22.5%	Difficulty related to technology management	25.7%	
4. I had difficulties connecting to the internet during classes.	Strongly Disagree	54.5%	Strongly Disagree	56.6%	
5. I had difficulties seeing the teacher on the screen during classes.	Strongly Disagree	72.7%	Strongly Disagree	66.7%	
6. I had no difficulty hearing the teacher during classes.	Strongly Disagree	72.7%	Partially Disagree	60.0%	
7. I could hear the music during the classes.	Strongly Agree	84.8%	Strongly Agree	80.0%	
8. *Which options best characterize how you attended the classes?	Alone	90.9%	Alone	86.7%	0.369
9. I needed assistance from someone (family/caregiver/friend) to perform movements during the classes, such as standing up from the chair.	Never needed help	100%	Never needed help	96.7%	0.476
10. The instructions provided by the teacher during the classes were difficult to understand.	Strongly Disagree	78.7%	Strongly Disagree	90.0%	
11. I felt safe during the classes.	Strongly Agree	81.8%	Strongly Agree	83.3%	
12. Overall, I did not have difficulty participating in the online program.	Strongly Agree	72.7%	Strongly Agree	63.3%	
13. I received the information from the research team to complete the online program successfully.	Strongly Agree	93.9%	Strongly Agree	100%	
14. My environment was suitable for performing the proposed activities during classes.	Strongly Agree	60.6%	Strongly Agree	70.0%	
Score (questions 1; 3 to 7; 9 to 14): 0–48	4.5 (3 – 6.1)		6.3 (4.6 – 7.9)		0.134

**SECTION II - ENJOYMENT AND ENGAGEMENT**					

15. I did not perform well during classes.	Partially Agree	33.3%	Partially Disagree	33.3%	
16. I felt my emotions settled down after participating in the online program classes.	Strongly Agree	54.5%	Strongly Agree	50.0%	
17. I think the online program classes brought me benefits.	Strongly Agree	75.7%	Strongly Agree	86.7%	
18. I can move myself easier after participating in the online program classes.	Strongly Agree	54.5%	Strongly Agree	63.3%	
19. My balance improved after participating in the online program classes.	Strongly Agree	51.5%	Neither Agree nor Disagree	36.7%	
20. I feel more confident walking around the house after participating in the online program classes.	Strongly Agree	60.6%	Strongly Agree	46.7%	
21. After participating in the online program classes, I feel more confident to bend down and pick up an object on the floor.	Strongly Agree	45.4%	Strongly Agree	53.3%	
22. I feel more confident sitting and standing up after participating in the online program classes.	Strongly Agree	57.5%	Strongly Agree	53.3%	
23. I feel more confident to turn 360º around an object after participating in the online program classes.	Strongly Agree	39.3%	Strongly Agree	36.7%	
24. My mood improved after participating in the online program classes.	Strongly Agree	60.6%	Partially Agree	46.7%	
25. My sleep quality improved after participating in the online program classes.	Strongly Agree	24.2%	Neither Agree nor Disagree	43.3%	
26. I can move my hands and fingers better during my daily activities after participating in the online program classes.	Strongly Agree	51.5%	Strongly Agree	50.0%	
27. A lot of attention and focus was required to perform the activities proposed during classes.	Strongly Disagree	66.6%	Strongly Disagree	50.0%	
28. I improved my memorization ability and level of attention/focus after participating in the online program classes.	Strongly Agree	51.5%	Partially Agree	46.7%	
29. The music motivated me during the classes.	Strongly Agree	87.8%	Strongly Agree	63.3%	
30. I had difficulty implementing the online program classes into my daily exercise routine.	Strongly Disagree	51.5%	Strongly Disagree	56.7%	
31. I felt engaged and motivated during the online program classes.	Strongly Agree	75.7%	Strongly Agree	80.0%	
32. I believe the classes were fun and joyful.	Strongly Agree	87.8%	Strongly Agree	83.3%	
33. I would participate in this online program in the future.	Strongly Agree	87.8%	Strongly Agree	90.0%	
Score (questions 15 to 33): 0–72		13.5 (10.1 – 16.9)		14.6 (11 – 18.2)	0.648

**SECTION III - SOCIALIZATION**					

34. Which of the figures below best describes your relationship with the instructor/therapist? (S =You; I = Instructor)^*^ 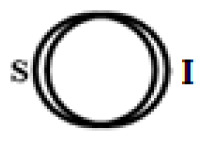	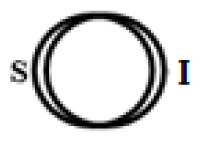	39.4%	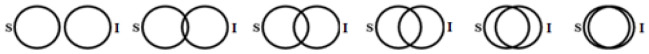	36.7%	
35. Which of the figures below best describes your relationship with your colleagues/group? (S = You; G = Group)^*^ 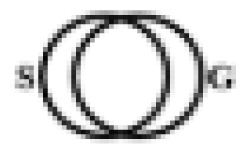	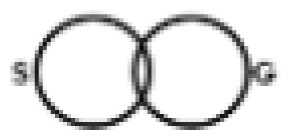	30.3%	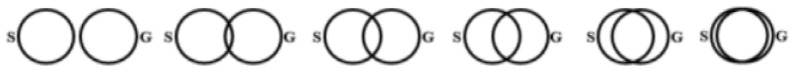	26.7%	
Total questions score (0–120 points)		18 (13.9 – 22.1)		20.9 (16.6 – 25.2)	0.345

*Note.* Categorical variables are presented as frequency (n) and percentage (%), and continuous variables as mean (95% confidence interval [95% CI]). Group comparisons were performed using the chi-square test or Fisher’s exact test for categorical variables and generalized linear models (GLM) for continuous variables. A significance level of 0.05 was adopted.

## Data Availability

The data supporting the findings of this study are available upon reasonable request from the corresponding author.
